# The effects of self-management interventions on depressive symptoms in adults with chronic physical disease(s) experiencing depressive symptomatology: a systematic review and meta-analysis

**DOI:** 10.1186/s12888-021-03504-8

**Published:** 2021-11-20

**Authors:** Lydia Ould Brahim, Sylvie D. Lambert, Nancy Feeley, Chelsea Coumoundouros, Jamie Schaffler, Jane McCusker, Erica E. M. Moodie, John Kayser, Kendall Kolne, Eric Belzile, Christine Genest

**Affiliations:** 1 Ingram School of Nursing, McGill University, Montreal, Canada; 2 St. Mary’s Research Centre, Montreal, Canada; 3 Centre for Nursing Research, Montreal, Canada; 4 Healthcare Sciences and e-Health, Department of Women’s and Children’s Health, Uppsala University, Uppsala, Sweden; 5 Epidemiology, Biostatistics and Occupational Health, McGill University, Montreal, Canada; 6 Department of Epidemiology, Biostatistics, and Occupational Health, McGill University Montreal, Montreal, Canada; 7 CIUSSS du Centre-Sud-de-l’Île-de-Montréa, Montreal, Canada; 8 Trillium Lakelands District School Board, Lindsay, Canada; 9 Faculty of Nursing Sciences, Université de Montreal, Montreal, Quebec Canada

**Keywords:** Self-management, Chronic disease, Depression, Anxiety, Decision-making, Systematic review

## Abstract

**Background:**

Chronic diseases are the leading cause of death worldwide. It is estimated that 20% of adults with chronic physical diseases experience concomitant depression, increasing their risk of morbidity and mortality. Low intensity psychosocial interventions, such as self-management, are part of recommended treatment; however, no systematic review has evaluated the effects of depression self-management interventions for this population. The primary objective was to examine the effect of self-management interventions on reducing depressive symptomatology in adults with chronic disease(s) and co-occurring depressive symptoms. Secondary objectives were to evaluate the effect of these interventions on improving other psychosocial and physiological outcomes (e.g., anxiety, glycemic control) and to assess potential differential effect based on key participant and intervention characteristics (e.g., chronic disease, provider).

**Methods:**

Studies comparing depression self-management interventions to a control group were identified through a) systematic searches of databases to June 2018 [MEDLINE (1946 -), EMBASE (1996 -), PsycINFO (1967 -), CINAHL (1984 -)] and b) secondary ‘snowball’ search strategies. The methodological quality of included studies was critically reviewed. Screening of all titles, abstracts, and full texts for eligibility was assessed independently by two authors. Data were extracted by one author and verified by a second.

**Results:**

Fifteen studies were retained: 12 for meta-analysis and three for descriptive review. In total, these trials included 2064 participants and most commonly evaluated interventions for people with cancer (*n* = 7) or diabetes (*n* = 4). From baseline to < 6-months (T1), the pooled mean effect size was − 0.47 [95% CI −0.73, − 0.21] as compared to control groups for the primary outcome of depression and − 0.53 [95% CI −0.91, − 0.15] at ≥ 6-months (T2). Results were also significant for anxiety (T1) and glycemic control (T2). Self-management skills of decision-making and taking action were significant moderators of depression at T1.

**Conclusion:**

Self-management interventions show promise in improving depression and anxiety in those with concomitant chronic physical disease. The findings may contribute to the development of future Self-management interventions and delivering evidence-based care to this population. Further high-quality RCTs are needed to identify sources of heterogeneity and investigate key intervention components.

## Background

### Prevalence and impact of depression in adults with chronic disease(s)

Depression affects 300 million people and is currently the leading cause of mortality worldwide substantially impacting social and occupational functioning [1–4]. Prevalence of depression is more common among individuals with chronic physical diseases [2, 3, 5]. Estimates indicate that approximately 20% of people with chronic physical diseases experience depression, at least twice the rate found in the general population [5–7].

Chronic diseases are increasingly prevalent and are currently estimated to account for 60% of all deaths worldwide [8, 9]. Studies have demonstrated that depression has a significant impact on the course and health outcomes of concomitant chronic physical diseases and complicates treatment [5, 6]. For example, depression has been shown to amplify somatic symptoms and diminish self-efficacy of health-related behaviours [6, 10, 11]. Furthermore, in addition to major depressive disorder, sub-threshold depression is associated with negative outcomes including increased morbidity in those with co-occurring physical diseases, and is also a risk factor for major depression [12–14].

### Self-management interventions for depression

It is recognized that timely treatment of depression is likely to improve functional ability, quality of life, and will considerably reduce the burden of chronic physical disease on the care recipient [15, 16]. However, worldwide less than half of those affected by depression receive adequate treatment, as there is often limited care available or barriers to accessing it, particularly high cost and lack of mental health professionals [12, 17, 18]. In response to the need for more depression support, one cost-efficient option that has shown promise is self-management interventions. Self-management interventions for depression have been formally incorporated into many healthcare systems and are recommended in best practice guidelines [19–21]. The only guidelines specifically targeting the treatment of depression in those with concomitant chronic physical disease(s), recommend self-management interventions for the treatment of mild to moderate depressive symptoms or as adjunctive therapy in the case of more severe symptoms [22]. However, to our knowledge, no systematic review has evaluated the effects of depression self-management interventions across chronic physical disease populations.

Self-management refers to the tasks an individual must undertake to live well with their chronic disease(s) [23, 24]. Generally, these tasks involve: a) medical management; b) maintaining, changing, or developing new meaningful behaviours; and c) dealing with the emotional impacts of the disease(s) [25]. In self-management, the individual’s role in their own care is considered central and the individual takes on the responsibility, as much as possible, of developing the necessary skills to manage their symptoms.

Self-management includes more than providing health or disease-related information, rather it is focused on behaviour change through the development of the skills and confidence needed for successful self-management of chronic disease [26, 27]. This is usually accomplished through the use of psychoeducational or behaviour strategies [28]. Learning these self-management skills can be done independently or in collaboration with health care professionals (HCPs) or a non-professional, often peer, support person [25, 29].

Arguably the most robust conceptualization of self-management is that articulated by Drs. Lorig and Holman based on nearly 25 years of work in the area [25, 30, 31]. This theoretically informed research indicates that self-management involves core skills (e.g., problem-solving, decision-making) that can be applied across chronic diseases [25, 32, 33]. From this perspective, self-management is not considered disease specific, but is focused on the development of broad skills, and self-management programs may therefore include people with a variety of chronic diseases [32, 33]. Additional skills may also be targeted when supporting the learned management of particular diseases. Aligned with this, based on existing literature, self-management skills specific to depression have been identified including behavioural activation (e.g., increasing positive activities) and social support (see Appendix A for details and references).

### Self-management, self-help, cognitive behavioural therapy, and self-care

A number of terms are often incorrectly used interchangeably with self-management, including self-help, cognitive behavioural therapy (CBT), and self-care. In comparison to self-management self-help encompasses a much broader range of interventions that are primarily self-directed (e.g., books, smartphone application). Self-help interventions are predominantly designed to limit contact with HCPs [34]. On the other hand, self-management interventions focus on specific skills and are often conducted in close collaboration with HCPs, although some may be self-directed [25]. Though many self-management interventions use principles of cognitive behavioural therapy (CBT), these are also distinct [29]. Self-management is one of the essential elements of the Chronic Care Model (CCM), an evidence-based guide to chronic disease management in primary care [26, 35]. Self-management interventions employ psychoeducational and/or behavioural strategies to assist care recipients and families develop the skills and confidence necessary to live well with a chronic disease (e.g., learning to locate resources, forming partnerships with HCPs) [25, 36]. Self-management is not a form of psychotherapy and rather than delivered by a therapist, self-management can be delivered by a variety of HCPs (e.g., it not a protected act) or as a self-directed intervention.

Finally, self-care is also frequently used interchangeably with self-management; however, they may be delineated on several fronts. Self-care involves managing one’s health, with or without a chronic condition. It also encompasses a broad range of strategies including ‘doing nothing.’ Another marked difference is that self-care does not usually involve HCP support and focuses primarily on health promotion or the prevention of disease or accident [37]. In contrast, as an element of the CCM, self-management was developed specifically for people with chronic disease and centres on the development of evidence-based skills [25, 28, 37].

## Aims of this Review

Accounting for the issues outlined above, the primary objective of this review is to determine the effects of self-management interventions on reducing depressive symptomatology among adults with chronic physical disease(s) and co-occurring depressive symptoms. The secondary objectives are:

- To determine whether the effects of self-management interventions on the primary outcome of depressive symptoms vary depending on the participants’ co-occurring chronic physical disease and baseline level of depressive symptoms (e.g., mild, moderate).
- To assess whether the interventions reviewed have differential effects on the primary outcome of depressive symptoms depending on intervention characteristics and content: the type and number of self-management skills targeted, mode of delivery (e.g., face-to-face, online), type and intensity of guidance, intervention provider, format (e.g., group or individual), and duration (minutes of participation) and length of time over which the intervention was delivered.
- To assess the effects of self-management interventions on other physiological and psychosocial outcomes (e.g., quality of life, fatigue) among the target population.

## Methods

### Methodological framework

The methods for this review were developed following the recommendations of the *Cochrane Handbook for Systematic Reviews of Interventions* [38] and the Preferred Reporting Items for Systematic Reviews and Meta-Analyses (PRISMA) statement [39]. The protocol was registered with the prospective register of systematic reviews (PROSPERO CRD42019132215).

### Criteria for considering studies for this review

#### Types of studies

Published or in press peer-reviewed full-text randomized controlled trials (RCTs) as well as cross-over trials and studies using a quasi-experimental design (e.g., controlled trials without randomization, but with a comparison group) were considered for inclusion in this review. To be eligible, the primary outcome (depressive symptoms) had to be measured pre- and post-intervention and only English and French language articles were included. Conference abstract and theses were excluded.

#### Types of participants and settings

Based on Lorig and Holman’s [25] conceptualization of self-management, participants with various chronic diseases were included. The target population for this review was adults (over age 18) with chronic physical disease(s) experiencing at least mild depressive symptoms according to a validated scale or clinical interview. The following psychometrically validated questionnaires and cut-off scores indicating at least mild depressive symptoms were eligible for inclusion:

- Beck Depression Inventory (scores ≥10) [40, 41]
- Beck Depression Inventory-II (scores ≥13) [42]
- Centre for Epidemiological Studies – Depression (scores ≥16) [43, 44]
- Centre for Epidemiological Studies – Depression 10-item version (scores ≥10) [45, 46]
- Hamilton Depression Rating Scale – Depression subscale (scores ≥8) [47]
- Hospital Anxiety and Depression Scale – Depression (scores ≥8) [48]
- Patient Health Questionnaire (scores ≥5) [49]
- Geriatric Depression Scale (scores ≥11) [50]

In terms of clinical interviews, the DSM-III (Diagnostic and Statistical Manual of Mental Disorders) and above [51–55] as well as the ICD-9 (International classification of diseases) and above [56, 57] were considered eligible.

Studies including participants with one or multiple chronic physical diseases as defined by the Public Health Agency of Canada (excluding dementia) and World Health Organization were included [9, 58]. Participants taking anti-depressant medication were eligible, if pharmacotherapy was not administered as part of the intervention being evaluated. In terms of co-morbidities, studies including participants with a diagnosis of bipolar disorder, post-partum depression, seasonal affective disorder, or post-traumatic stress disorder were excluded as the etiology, disease course, and treatment recommendations for these conditions differ from depression [59–62]. No limits were placed on the setting in which the study was conducted.

#### Types of interventions

##### Experimental interventions.

Studies evaluating self-management interventions for depressive symptoms were included. Interventions were eligible if they incorporated at least one of the key self-management skills described in Appendix A. These skills were derived from the theoretical literature as well as from existing self-management interventions from reputable (peer-reviewed or government issued) sources [25, 29, 63–65]. Further, the intervention had to be administered to the individual directly. Interventions in which the person with the chronic disease participated with someone else (e.g., family member) were excluded due to the confounding effects of social support [66].

Multi-component interventions were eligible as a long as the above self-management criteria were met and at least one component targeted mood, distress, or depression. To avoid confounding effects, studies evaluating interventions including a component in which pharmacotherapy was administered were excluded. All formats of interventions were eligible (e.g., workbooks, online modules). Level of guidance by HCPs was not an exclusion criterion; self-directed, minimal contact, and HCP administered interventions were all eligible.

##### Comparator interventions.

Eligible control comparisons were no treatment, treatment as usual, waitlist, and attention control groups, as long as the participants were not receiving active components of the interventions (e.g., psychological therapy or the self-management skills outlined in Appendix A).

#### Outcome measures

The primary outcome was depressive symptoms. The secondary outcomes were a) improvement in psychosocial outcomes such as quality of life, anxiety, self-management skills, and self-efficacy; b) improvement in physical health measures; c) improvement in alcohol and drug consumption; and/or d) decrease in health care utilization.

### Information sources and study selection

#### Search strategies

Eligible studies were primarily identified through searches of electronic bibliographic databases. Secondary search strategies consisted of verifying the reference lists of the included full-texts and using the PubMed ‘find similar’ function. Unpublished findings were also sought through the Cochrane library as well as the national and international trial registries outlined in the *Cochrane Handbook for Systematic Reviews* [38]. These were searched for relevant trial protocols and, if published findings of these trials could not be found, authors were contacted directly for further information.

#### Database searches

Eligible studies were identified by searching the following databases: MEDLINE (1946-), EMBASE (1996 -), PsycINFO (1967 -), and Cumulative Index to Nursing & Allied Health (CINAHL) (1984 -). The search was conducted in June 2018 and no limits were applied. All databases were searched using a combination of keywords and subject headings across three concepts: a) self-management, b) depression, and c) trial design (RCT or quasi-experimental). The search strategy was developed in consultation with a health sciences librarian and were assessed using the Peer Review for Electronic Search Strategies (PRESS) guidelines [67]. The full primary electronic search for Ovid Medline is included in Appendix B. All titles and abstracts were downloaded to a citation manager, EndNote, and screened using Rayyan online software. Duplicates were removed according to the procedures outlined in Bramer, Giustini [68].

#### Study selection

Two authors independently assessed the eligibility of all retrieved titles, abstracts, and full texts to confirm inclusion or exclusion. Two authors also separately examined the reference lists of included full texts. Finally, searches were conducted using the ‘find similar’ function of PubMed. Any disagreements were discussed with a third author until consensus was established. Multiple reports relating to the same study were aggregated so that findings reflected each study rather than each report.

### Data extraction

Data were extracted using a standardized Microsoft Excel form that was developed based on the *Cochrane Handbook for Systematic Reviews* [38]. The form was adapted from one used in a previous systematic review conducted by team members [69, 70]. Data were extracted by one author and confirmed by at least one other. Disagreements were discussed with a third author until resolved.

The following data on study characteristics were extracted: citation details, country of origin, study design, aims, theoretical framework, population (age, diagnosis, gender, depressive symptoms), sample size, setting (e.g., hospital, community), summary of intervention and control groups, self-management skills in the intervention, format of the intervention, intervention provider, level of guidance, duration of the intervention (number of minutes of participation), length of intervention (time period over which the intervention was delivered, e.g., 2-months of sessions), monitoring of fidelity and adherence, outcomes, timing of measurement, and attrition [38]. Outcomes were grouped into two time periods: T1 from baseline < 6-months post-baseline and T2 ≥ 6-months post-baseline. If any data were missing or unclear, the authors of the manuscript were contacted for further information.

#### Methodological quality

Two authors (combination of the first, seventh, and eight authors) independently assessed the risk of bias of each study based on the criteria outlined in the Cochrane Risk of Bias tool [71]. Again, disagreements were discussed with a third author until consensus was reached. The risk of bias was evaluated according to the following criteria: a) inclusion criteria specified, b) pre-specified primary outcome(s), c) psychometric properties of primary outcomes provided, d) explicit power calculation, e) target sample size reached, f) appropriate randomization procedures and allocation concealment, g) discussion of potential co-interventions, h) baseline characteristics of all groups provided, i) blinding of outcome assessors, participants, and interventionists, j) adherence to intervention (> 75%), k) fidelity monitoring, l) management of missing data (intention-to-treat analysis), m) participant retention (> 80%), and n) reasons for attrition stated.

Each potential source of bias was evaluated as having been met (score 1) or not met (score 0). If the information was not specified in the manuscript the authors were contacted. If the information could not be clarified, the item was deemed not to have been met. Studies were considered to be of high methodological quality if 13–17 of the criteria were met, moderate quality if 8–12 were met, and low fewer than 8 were met. Direct quotes from each study as well as supporting comments were included in the evaluation of each. For evidence of selective reporting, study protocols or trial registration of included articles, when available, were compared to the published findings and unexplained discrepancies were noted [71].

### Data analysis

Effect sizes (Hedge’s adjusted g) were calculated using outcome scores at post-intervention assessment between treatment and control conditions for the T1 and T2 time periods [38, 72]. Initially analyses were planned for three time periods: T1< 3 months post-baseline, T2 - 3 to < 6 months post-baseline, and T3 ≥ 6 months post-baseline. Due to the limited sample size, T1 and T2 were combined. Hedge’s adjusted g was selected to reduce potential bias due to small sample sizes [73]. The magnitude of the effect size can be interpreted according to the benchmarks outlined by Cohen (1988) [74]; namely, small (0.2), moderate (0.5), and large (0.8).

Pooled mean effect sizes were calculated to obtain a summary statistic for the T1 and T2 periods. If a study reported both per protocol and intention-to-treat analyses, per protocol data were included in the meta-analysis. When outcome data were collected at more than one time point within the timeframe, as most studies reported data at 6-months, the data collection closest to this point was used (e.g., if 6 and 12 month post-baseline data were available, 6 month data were entered into the meta-analysis). The Higgin’s statistic (I^2^) was calculated to measure the heterogeneity across studies and interpreted as 0% indicating no heterogeneity, 25% low, 50% moderate, and > 75% high heterogeneity [71]. It was anticipated that there would be considerable heterogeneity across studies arising from differences in the interventions delivered, sample characteristics, and study designs. As such, a random effects model was used for meta-analysis calculations. As the number of studies was small, in addition to the DerSimonian–Laird approach, the Knapp-Hartung approach was used to make small-sample adjustments to the variance estimates of any outcomes that were statistically significant [75]. This more conservative approach produces a wider confidence limit appropriate when the sample size is small [75]. All tests were two-sided, and the significance level was set at *p* < 0.05. These analyses were conducted using RevMan 5.3.

Publication bias was evaluated through inspection of funnel plots of the primary outcome variable of depression [38]. The effect of potential moderators on the primary outcomes was also assessed [76]. The prespecified moderators, participant and intervention characteristics, are detailed in Table 1. Studies were separated into sub-groups based on these variables and meta-regressions were performed to identify whether there was a statistically significant difference in outcomes between the sub-groups. There is no consensus on the number of studies required to run a meta-regression; these were performed when sub-groups included 4 or more studies (*p*-value < 0.05 was set to establish significance) [77]. As meta-regressions cannot be performed using the Revman 5.3 software, these were calculated using the ‘metareg’ program in STATA (version 15.1). If needed information for any of the above calculations was not reported in the publication, authors were contacted for further details. If data required for inclusion in the meta-analysis were not available, the study was included for descriptive review only. Due to the substantial diversity in reported outcomes, only outcomes reported at least three times at one time point (T1 or T2) were included for review.

**Table 1:** Descriptive summary of included studies

Author, Year, Country, Quality Assessment Score (QAS) (/17)	Aim(s)	Demographics	Intervention and control conditions and assessments	Outcome(s) [Primary (P), Secondary (S), Unspecified (O)]
Barley et al., 2014 [78, 79] United Kingdom Pilot RCT (2 groups) QAS:11	To explore the acceptability and feasibility of procedures to inform a definitive RCT of a practice nurse-led personalised care intervention for CHD patients with at least probable depression and chest pain.	**Symptomatic chronic heart disease (with active chest pain)** *N* = 81 (T = 41, C = 40) Mean age: 65 (SD = 11) % female = 35.8 Race/ethnicity: 83% white Mean HADS-D score: T = 12 (SD = 3), C = 11 (SD = 3)	**T:** Nine sessions (one face-to-face assessment + 15-min follow-up phone calls) with nurse focused on identifying problems contributing to depression, providing support resources, devising personal health plan, goal setting, and building self-efficacy. **C:** Usual care. **Format:** Individual. **Mode of delivery:** Face-to-face and telephone. **Interventionist:** Nurse. **Intervention duration:** Mean 203 min (SD 100) of nurse time (mean 78min SD 19 for face-to-face assessment; mean 125min SD 91 in follow-up telephone calls). **Intervention length**: 6-months. **Level of guidance**: Guided **Timing of measures**: 1-, 6-, and 12-months post baseline.	**P:** T1 = C for depression T2 = C depression **S:** T1 = C for anxiety T1 = C for MCS and PCS T2 = C for anxiety, MCS, and PCS
Boele et al., 2018 [80, 81] Netherlands RCT (3 groups) QAS: 10	To decrease depressive symptoms using low-intensity guided self-help based on problem-solving therapy delivered online to increase accessibility and decrease barriers to accessing mental health care.	**Glioma (CNS cancer)** *N* = 115 (T = 45, C1 = 26, C2 = 44) Mean age: T = 43.6 (SD = 11.7), C1 = 52.8 (SD = 9.3), C2 = 46.4 (SD = 12.3) % female: T = 57.8, C1 = 65.4, C2 = 59.1 Most common diagnosis in C1: non-Hodgkin lymphoma (46.2%) Mean CES-D score: T = 21.5 (SD = 6.1), C1 = 25.1 (SD = 6.7), C2 = 24.1 (SD = 6.6)	**T** = Guided self-help course based on problem-solving therapy including disease specific information. Five modules and exercises. Online support and feedback on exercises provided by coach. **C1** = Non-CNS cancer control group. Received intervention. **C2** = Glioma 12-week waitlist control group (WLC). **Format:** Individual. **Mode of delivery:** Online **Interventionist:** Psychologist, nurse, or psychology student (coaches). **Intervention duration:** n/a **Intervention length**: 5 weeks. **Level of guidance**: Guided self-directed. **Timing of measures**: 1.5-, 3-, 12-months post-baseline (last outcome measure not included for analysis as WLC group had completed intervention).	**P:** T1 = C2 for depression **S:** T1 > C2 for MCS (ES: 0.87) T1 = C2 for PCS
Espahbodi et al., 2015 [82] Iran Quasi-experimental (randomized matched design) QAS: 6	To investigate the impacts of education on psychological symptoms (anxiety and depression) in patients undergoing dialysis.	**Renal Failure (receiving dialysis)** *N* = 55 (T = 27, C = 28) Mean age: T = 49.1 (SD = 14.5), C = 52.3 (SD = 15.6) % female: T = 52, C = 50 Mean HADS-D: T = 10.2 (SD = 3.4), C = 10.1 (SD = 3.4)	**T:** Psychoeducational intervention (3 sessions × 60 min) focused on disease-specific information (e.g., physiology, causes, treatments) as well as problem-solving, stress management, adaptive responses, and muscle relaxation. **C**: Usual care. **Format:** Group **Mode of delivery:** Face-to-face. **Interventionist:** Unspecified. In collaboration with a nephrologist and psychiatrist. **Intervention duration:** 180 min. **Intervention length**: Approximately 5 days. **Level of guidance**: Guided. **Timing of measures**: 1-month post-baseline.	**P:** T1 = C for depression and anxiety
Fischer et al., 2015 [83] Germany RCT (2 groups) QAS: 9	To evaluate the feasibility and efficacy of a fully automated internet-based CBT program to reduce depressive symptoms in patients with multiple sclerosis (MS).	**Multiple sclerosis** *N* = 90 (T = 45, C = 45) Mean age: T = 45.4 (SD = 12.6), C = 4524 (SD = 10.6) % female: T = 76, C = 80 Mean BDI score: T = 19.4 (SD = 9.0), C = 18.4 (SD = 8.2)	**T** = Ten online modules using simulated dialogue and tailored based on participant response. Content draws on: 1) behavioral activation, 2) cognitive modification, 3) mindfulness and acceptance, 4) interpersonal skills, 5) relaxation, physical exercise and lifestyle modification, 6) problem solving, 7) childhood experiences and early schemas, 8) positive psychology interventions, 9) dreamwork and emotion-focused interventions, and 10) psychoeducation. **C** = 2.25-month WLC **Format:** Individual. **Mode of delivery:** Online. **Interventionist:** Self-directed. **Intervention duration:** Self-directed. Mean use: 332 min (range 50–905 min). **Length of intervention**: 2.25 months. **Level of guidance**: Self-directed. **Timing of measures**: 2.25- and 8.25-months post-baseline (last outcome measure not included for analysis as WLC group had completed intervention).	**P:** T1 = C for depression **S:** T = C for fatigue
Lamers et al., 2010a [84, 85] Netherlands RCT (2 groups) QAS: 12	To evaluate the effectiveness of a nurse-administered minimal psychological intervention in reducing depressive symptoms in elderly primary care patients with type II diabetes or COPD with co-morbid non-severe depression and examine whether type of chronic illness modified the effects of the intervention.	**Type II diabetes, COPD** *N* = 361 (T = 183, C = 178) Mean age: T = 70.8 (SD = 6.5), C = 70.6 (SD = 6.8) % female: T = 46.4, C = 46.6 Primary diagnosis: T: 49.7% diabetes, 50.3% COPD C: 52.8% diabetes, 47.2% COPD Mean BDI score: T = 17.1 (SD = 7.2) C = 17.7 (SD = 8.0)	**T** = Tailored intervention with variable number of sessions (2–10) based on principles of self-management and CBT and includes 5 phases: 1) exploring feelings, cognitions, and behaviours, 2) mood, symptom, and behaviourmonitoring, 3) linking mood to behaviour, 4) action planning, and 5) evaluation of progress in achieving goals. **C** = Usual care. **Format:** Individual. **Mode of delivery:** Face-to-face. **Interventionist:** Nurse. **Intervention duration:** Mean 240 min. **Intervention length**: Tailored up to 3-months. **Level of guidance**: Guided. **Timing of measures**: Approximately 3.25-, 6-, and 12-months post-baseline (assuming 3-month intervention period).	**P:** T1 = C for depression T2 = C for depression **S:** T1 = C for MCS and PCS T2 = C for MCS and PCS COPD sub-group* T1 = C for MCS T2 = C for MCS DMII sub-group* T1 = C for MCS T2 = C for MCS (note: T>C for MCS at 9-months post intervention completion)*
Lamers et al. 2010b [86] Note: Subgroup analysis of Lamers 2010a Netherlands RCT (2 groups)	To evaluate the effect of a nurse-administered minimal psychological intervention on disease specific quality of life, depression, and anxiety in elderly primary care patients with COPD with co-morbid non-severe depression.	**COPD** *N* = 187 (T = 96, C = 91) Mean age: T = 70.5 (SD = 6.3), C = 71.5 (SD = 7.1) % female: T = 38.5, C = 41.8 Mean BDI score: T = 17.1 (SD = 6.5), C = 18.3 (SD = 7.2)	See Lamers et al., 2010a	**P:** T1 = C for depression* T2 = C for depression* (Note: T>C for depression at 9-months post intervention completion).* S: T1=C for anxiety* T2=C for anxiety* (Note: T>C for anxiety at 9-months post intervention completion)*
Lamers et al., 2011 [87] Note: Subgroup analysis of Lamers 2010a Netherlands RCT (2 groups)	To evaluate whether a nurse-administered minimal psychological intervention based on CBT and self-management principles improves disease-specific quality of life and glycemic control in patients with type II diabetes and co-morbid non-severe depression.	**Type II diabetes** *N* = 208 (T = 105, C = 103) Mean age: T = 70.7 (SD = 6.6), C = 69.7 (SD = 6.6) % female: T = 51.4, C = 50.4 Depression level: Not specified. Participants underwent Mini International Neuropsychiatric review. Those with minor depression, mild-to-moderate major depression or dysthymia were included.	See Lamers et al., 2010a	**S:** T1 = C for glycemic control (HbA1c) T2 = C for glycemic control (HbA1c)
Lee et al., 2014 [88] Republic of Korea Quasi-RCT – group allocation based on consent date (2 groups) QAS: 12	To evaluate the effectiveness of a tablet PC-based single session psychoeducation intervention for cancer patients reporting significant levels of distress.	**Type II diabetes** *per group data not available *N* = 36 (T = 19, C = 17) Median age: 57.5 (range 34–71) % female: 55.6 Mean HADS-D score: T = 12.0 (SD = 3.7), C = 12.7 (SD = 1.5)	**T** = Twenty-minute psychoeducation video clip. Content consisted of distress education, cancer survivor interview, coping strategies and stress management, and psychological services. **C** = Control movie clip of scenic images and relaxing music. **Format:** Individual. **Mode of delivery:** Video presented on computer tablet. **Interventionist:** n/a **Intervention duration:** 20-min. **Length of intervention**: 20-min. **Level of guidance**: Self-directed **Timing of measures**: 1-day (post-intervention same day as baseline measures) and 2–4 weeks post-baseline.	**P:** T1 > C for depression (ES: − 1.13) and MCS (ES: 1.08) T1 = C for anxiety
Moncrieft et al., 2016 [89] United States RCT (2 groups) QAS: 9	To determine the effect of a multicomponent behavourial intervention on weight, glycemic control, renal function, and depressive symptoms in adults with DMII and depressive symptoms.	**Cancer patients receiving chemotherapy treatment** *N* = 111 (T = 57, C = 54) Mean age: T = 54.8 (SD = 8.3), C = 54.8 (SD = 6.3) % female: T = 64.9, C = 77.8 Mean BDI-II score: T = 19.3 (SD = 7.1) C = 21.2 (SD = 7.1)	**T:** Structured lifestyle intervention (17 sessions × 1.5–2 h). Two individual sessions followed by two weekly, four bi-weekly, and nine monthly group sessions. Intervention components focused on diet and physical activity, including a weight loss, exercise, and caloric intake goals, combined with cognitive behavioural and social learning approaches to managing depression. **C:** Usual care + brief educational booklet on diabetes management. **Format:** Individual and group. **Mode of delivery:** Face-to-face. **Interventionist:** Therapists. **Intervention duration:** 1530 to 2040 min. **Intervention length**: 12-months. **Level of guidance**: Guided. **Timing of measures**: 6- and 12-months post-baseline.	**P:** T2 > C for depression (ES: − 0.62) T2 = C for glycemic control (HbA1c)
Penckofer et al., 2012 [90] United States RCT (2 groups) QAS: 12	To examine the effects of a nurse-delivered psychoeducation intervention on depression, anxiety, and anger among women with type II diabetes.	**Type II diabetes** *N* = 74 (T = 38, C = 36) Mean age: T = 54.8 (SD = 8.8), C = 54.0 (SD = 8.4) % female: 100 Mean CES-D score: T = 27.7 (SD = 9.3) C = 28.9 (SD = 9.5)	**T** = Sessions [(8 weekly sessions + 2 booster sessions) × 1 h/session] focused on recognizing signs and symptoms of depression, relationship between mood, metabolic control, and self-care behaviours, the management of depression, anxiety, and anger using CBT. Includes elements from existing interventions such as CBT program for depression, progressive muscle relaxation CD, and system for management of anger including workbook and video. **C** = Usual care. **Format:** Group. **Mode of delivery:** Face-to-face. **Interventionist:** Nurse. **Intervention duration:** 600 min **Intervention length**: 6-months **Level of guidance**: Guided. **Timing of measures**: 3- and 6-months post-baseline.	**P:** T1 > C for depression (ES: − 0.78) T1 = C for trait anxiety T1 = C for state anxiety T2 > C for depression (ES: − 0.94) and trait anxiety (ES: − 0.62) T2 = C for state anxiety (ES: − 0.74) **S**: T1 = C for MCS, PCS, and glycemic control (HbA1c) T2 = C for PCS and glycemic control (HbA1c) T2 > C for MCS (ES: 0.60)
Rees et al., 2017 [91] Australia Pilot RCT (2 groups) QAS: 13	To provide preliminary evidence for the impact of problem-solving therapy for diabetes in adults with diabetic retinopathy and diabetes distress.	**Type II diabetes and diabetic retinopathy** *N* = 40 (T = 21, C = 19) Mean age: T = 60.1 (SD = 7.0), C = 59.6 (SD = 8.8) % female: T = 33.3, C = 31.6 Mean PHQ-9 score: T = 10.5 (SD = 5.2) C = 10.2 (SD = 5.7)	**T:** Provided publicly available information on diabetes + problem solving therapy for diabetes, which consisted of weekly sessions (8 × 45–60 min) in which participants identified problems related to diabetes and were guided through a problem-solving process. Participants were also asked to make plans to engage in enjoyable activities. **C:** Usual care + same publicly available brochures on diabetes as T group. **Format:** Individual **Mode of delivery:** Phone or in-person (based on preference and availability). **Interventionist:** Research assistant supervised by clinical psychologist. **Intervention duration:** 360–480 min. **Intervention length**: 2-months **Level of guidance**: Guided. **Timing of measures**: 3- and 6-months post-baseline.	**S:** T1 = C for depression and glycemic control (HbA1c) T2 = C for depression and glycemic control (HbA1c)
Schroder et al., 2014 [92] Germany RCT (2 groups) QAS: 10	To evaluate the feasibility and efficacy of an online program for depression in individuals with epilepsy and co-morbid depressive symptoms.	**Epilepsy** *N* = 78 (T = 38, C = 40) Mean age: T = 35.0 (SD = 10.0), C = 40.0 (SD = 11.9) % female: T=67.5, C=84.2 Mean BDI score: T = 22.2 (SD = 10.4) C = 19.4 (SD = 9.8)	**T:** Ten online modules (10–60 min each) comprised mostly of CBT elements (cognitive restructuring, behavioural activation) and mindfulness and acceptance exercises. **C:** 9-week WLC **Format:** Individual **Mode of delivery:** Online. **Interventionist:** Self-directed. **Intervention duration:** 100–600 min. **Intervention length**: 2.25 months. **Level of guidance**: Self-directed. **Timing of measures**: 2.25 months post-baseline.	**P:** T1 = C for depression
Sharpe et al., 2004 [93] United Kingdom (Scotland) Non-randomized matched control group design (2 groups) QAS: 12	To perform preliminary evaluation of the feasibility and efficacy of a nurse-led intervention with oncology outpatients.	**Cancer** (outpatients with breast, gynaecological, bladder, prostate, testicular and colorectal) *N* = 60 (T = 30, C = 30) Mean age: T = 58.0 (SD = 10.6), C = 56.0 (SD = 10.5) % female: T = 93.3, C = 93.3 Mean HADS-D score: T = 10.4 (SD = 3.6) C = 10.3 (SD = 4.0)	**T:** The intervention consisted of up to 10 weekly problem-solving therapy sessions (30 min each) to help with a positive and systematic approach to tackling problems, education about depression, encouragement to speak with their general practitioner about anti-depressant medication, and coordination and monitoring of the participant’s depression treatment. Participants could contact the nurse for further booster sessions. **C:** Usual care. **Format:** Individual. **Mode of delivery:** Face-to-face or phone. **Interventionist:** Nurse supervised by psychiatrist. **Intervention duration:** Nurse spent mean of 360 min with participants. **Intervention length**: Ranged from 0.5–4 months (with 6 participants requesting booster sessions). **Level of guidance**: Guided. **Timing of measures**: 3- and 6-months post-baseline.	**P:** T1 > C for depression (ES: − 0.87) T2 > C for depression (ES: − 0.58) **S**: T1 > C for anxiety (ES: − 1.25) T2 > C for anxiety (ES: − 0.88)
Sharpe et al., 2014 [94] **(SMaRT Oncology-2)** United Kingdom (Scotland) RCT (2 groups) QAS: 14	To compare the effectiveness of an integrated treatment programme for major depression in patients with cancer with usual care for patients with cancer who have co-morbid major depression and a survival prognosis of at least a year.	**Cancer with prognosis of survival over 12-months.** *N* = 500 (T = 253, C = 247) Mean age: T = 56.6 (SD = 10.0), C = 56.1 (SD = 10.2) % female: T = 90, C = 90 Mean SLC-20 score: T = 2.10 (SD = 0.62) C = 2.11 (SD = 0.56)	**T:** Based on Strong et al. (2008). Primary care physician and oncologist informed of major depression disorder diagnosis + multicomponent treatment program integrated into cancer care in which participants form relationships with nurses who provide information about depression, deliver problem-solving therapy, and monitor progress (up to 10 sessions X 45 min and additional sessions available for those not meeting treatment targets). **C:** Usual care + primary care physician and oncologist informed of major depression diagnosis + participant encouraged to consult their primary care physician to obtain treatment. **Format:** Individual. **Mode of delivery:** Primarily face-to-face, sometimes telephone. **Interventionist:** Oncology nurses supervised by a psychiatrist. **Intervention duration:** 405 min. Median number of sessions: 9 (range 0–10). **Intervention length**: 4-months for initial sessions and further sessions for those who are not meeting treatment targets. **Level of guidance**: Guided. **Timing of measures**: 3-, 6-, 9-, and 12-months post-baseline.	**P:** T1 > C for depression (ES: − 0.87) T2 > C for depression (ES: − 1.03) **S**: T1 > C for anxiety (ES: − 0.61) and fatigue (ES: − 0.41) T2 > C for anxiety (ES: − 0.71) and fatigue (ES: − 0.60)
Strong et al., 2008 [95] **(SMaRT oncology 1)** United Kingdom (Scotland) RCT (2 groups) QAS: 14	To assess the efficacy and cost of a nurse-delivered complex intervention designed to treat major depressive disorder in patients with cancer.	**Cancer** *N* = 200 (T = 101, C = 99) Mean age: T = 56.6 (SD = 11.4), C = 56.6 (SD = 12.3) % female: T = 69, C = 72 Median SCL-20 score (IQR): T = 2.35 (2.05–2.75), C = 2.25 (1.95–2.75)	**T:** Maximum of 10 session (45-min each) over 3-months followed by monthly monitoring of symptoms in the next 3-months and. optional 1–2 sessions for those whose depression scores increased. The intervention included education about depression and treatment, problem-solving treatment, and communicating with the participant’s primary care physician and oncologist about their depression diagnosis. **C:** Usual care + informed primary care physician and oncologist of depression diagnosis and, if requested, provided advice regarding choice of antidepressant medication. **Format:** Individual. **Mode of delivery:** Primarily in-person, some by telephone if needed. **Interventionist:** Oncology nurse supervised by a psychiatrist. **Intervention duration:** Mean of 315 min based on mean of 7 sessions (range 2–10). **Intervention length**: 6-months. **Level of guidance**: Guided. **Timing of measures**: 3-, 6-, and 12-months post-baseline	**P:** T1 > C for depression* T2 > C for depression* **S**: T1 > C for anxiety and fatigue* T2 > C for anxiety and fatigue*
Thorton et al., 2009 [96] United States RCT (2 groups) Secondary analysis QAS: 11	To test experimentally whether a psychological intervention reduces depression-related symptoms and markers of inflammation among cancer patients.	**Breast cancer** (Stage II/III, surgically treated, and waiting for adjuvant therapies) *N* = 45 (T = 23, C = 22) Mean age: T = 50.0 (SD = 8.6), C = 50.0 (SD = 11.6) % female: 100 Mean CES-D Iowa short-form score not reported. All participants included in the secondary analysis scored ≥10 as part of inclusion criteria.	**T:** Group sessions of 8–12 patients for 90 min for 18 weekly sessions followed by 8 monthly sessions. Topics included stress management, emotional distress, social adjustment, health behaviours (e.g., diet, exercise), and adherence to treatment. **C:** Usual care. **Format:** Group. **Mode of delivery:** Face-to-face (some telephone contact to catch up on information if sessions were missed). **Interventionist:** Psychologists. **Intervention duration:** 2340 min. **Intervention length**: 12-months. **Level of guidance**: Guided. **Timing of measures**: 4-, 8-, 12-months post-baseline.	**P:** T1 > C for depression* T2 > C for depression* **S**: T1 > C for fatigue* T2 > C for fatigue*
Walker et al., 2014 [97] **(SMaRT Oncology-3)** United Kingdom (Scotland) RCT (2 groups) QAS: 13	To assess the efficacy of an integrated treatment program for major depressive disorder in patients with lung cancer compared with usual care.	**Lung cancer** *N* = 142 (T = 68, C = 74) Mean age: T = 63.6 (SD = 8.8), C = 63.9 (SD = 8.7) % female: T = 65, C = 65 Mean SCL-20 score: T = 1.90 (SD 0.52), C = 1.98 (0.58)	**T:** Adapted from Sharpe et al. (2014). Maximum of 10 sessions (30–45 min) over 4-months followed by monitoring of symptoms and optional additional sessions for participants who did not meet treatment target. Nurses establish therapeutic relationship, provide information about depression, delivey problem-solving therapy and behavioural activation and monitor progress. Psychiatrists supervise treatment, advise primary care physicians, and provide direct consultation to participants not progressing. **C:** Usual care + primary care physician and oncologist informed of the diagnosis of major depression and participant encouraged to see primary care physician to obtain treatment. **Format:** Individual. **Mode of delivery:** Primarily face-to-face, some telephone contact. **Interventionist:** Nurse and psychiatrist. **Intervention duration:** 240–360 min (median number of sessions 8 IQR 7–10). **Intervention length**: 8-months. **Level of guidance**: Guided. **Timing of measures**: 1-, 2-, 3-, 4-, 5-, 6-, 7-, and 8-months post-baseline. Outcomes averaged over the participants time in the trial (up to 8-months).	Outcomes averaged over the participants time in the trial (up to 8-months). **P**: T > C for depression* **S**: T > C for anxiety* T = C for fatigue*

Notes: Only post-intervention primary and secondary outcomes of interest in this review reported across at least 3 studies within one time period (T1 and/or T2) included. T1- baseline to < 6 months post-baseline; T2 ≥ 6 months post-baseline. T = treatment condition; C = control condition; T > C = treatment significantly superior to control; T < C = control superior to treatment; T = C = no significant differences between. ES = Effect size (Hedge’s g calculated at 95% confidence level); Intervention duration = number of minutes spent participating in intervention based on reported participation or expected duration; Intervention length= length of time over which intervention was delivered; *Indicates that insufficient data available to calculate effect size so outcome is as reported by authors; sign of effect size based on negative orientation of scale (as intervention always compared with control – scales in which decreased scores indicate improvement are negative); Duration of the intervention based on reported mean or median adherence (in minutes) multiplied by the number of sessions, or, if not available, amount of time authors reported intervention would take (e.g., 4 sessions X 60 min = 240 min). If the range of individual sessions was provided (e.g., 15 to 30 min per session), the midpoint (e.g., 22.5) was multiplied by the number of sessions.; *IQR* interquartile range; *CHD* coronary heart disease, *CNS* central nervous system, *PST* problem-solving therapy; *WLC* wait list control group; *MS* multiple sclerosis; *COPD* chronic obstructive pulmonary disorder; *ER* emergency room; *QoL* quality of life; *CBT* cognitive behavioural therapy; *BDI* Beck Depression Inventory [40]; *BDI-II* Beck Depression Inventory-II [42]; *CES-D* Centre for Epidemiological Studies-Depression [44]; *HADS-D* Hospital Anxiety and Depression Scale-Depression [48]; *PHQ-9* Patient Health Questionnaire [49]; *HRQoL* Health related Quality of Life and includes: *PCS* physical health composite scale; *MCS* mental health composite scale

## Results

### Study selection

**Fig. 1:**
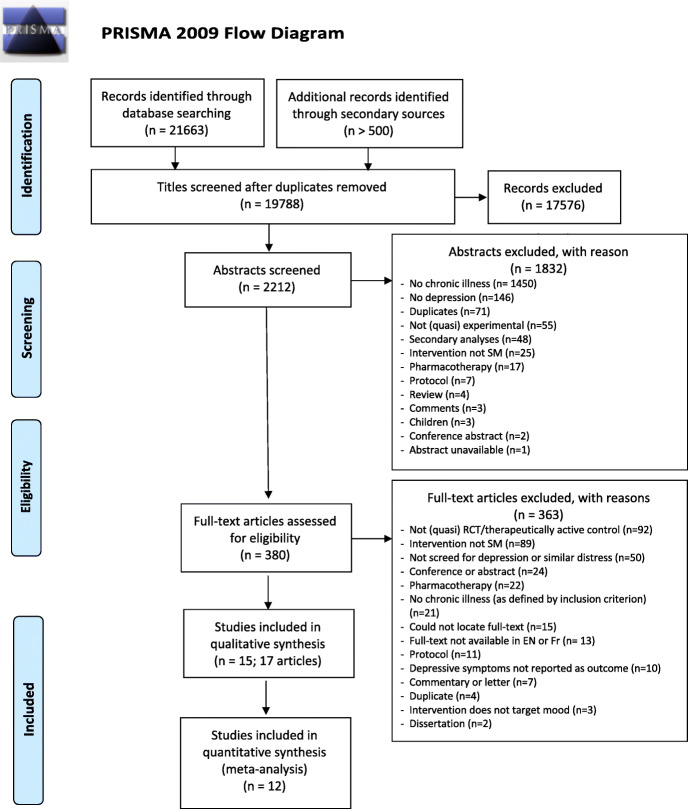
PRISMA 2009 Flow Diagram

In total, 21,663 titles were retrieved through database searches and over 500 titles were screened through secondary searches. After removing duplicates, 19,788 titles remained. Screening of these resulted in the inclusion of 2212 abstracts, 1832 of which were excluded, leaving 380 full texts to be reviewed. Seventeen manuscripts reporting on 15 studies were retained: 12 for inclusion in the meta-analysis and three for descriptive review only. Flow of studies and reasons for exclusion are detailed in Fig. 1.

### Description of studies

Characteristics of included studies are described in Table 2 and there was no indication of publication bias (Appendix C). Studies were conducted in the United Kingdom (*n* = 5), United States (*n* = 3), the Netherlands (*n* = 2), Germany (*n* = 2), Iran (*n* = 1), Republic of Korea (*n* = 1), and Australia (*n* = 1). Eleven studies used a 2-group RCT design (including two pilot trials), two studies used a 2-group quasi-experimental design, and one used a 3-group RCT design. The intervention groups were compared to usual care (*n* = 6), attention control groups (*n* = 6) (e.g., provided publicly available health information), or waitlist control (*n* = 3).

**Table 2:** Effect sizes for T1 and T2 for secondary outcomes

	Timepoints					
	T1			T2		
Secondary Outcomes	# of studies	SMD (95% CI)	I^**2**^ (%)	# of studies	SMD (95% CI)	I^**2**^ (%)
Anxiety	7	-0.42 [− 0.73, − 0.12]	73	4	− 0.52 [− 0.94, − 0.10]	77
Mental Component Score (HRQoL)	5	0.43 [0.09, 0.76]	60	3	0.12 [− 0.28, 0.53]	67
Physical Component Score (HRQoL)	5	0.01 [−0.18, 0.20]	0	3	0.03 [−0.18, 0.24]	0
Fatigue	3	−0.36 [− 0.67, − 0.06]	50	1	−0.60 [− 0.78, − 0.41]	
Glycemic Control (HbA1c)	3	− 0.08 [− 0.57, 0.41]	49	4	−0.35 [− 0.62, − 0.07]	0

Note: T1 baseline to < 6-months; T2 ≥ 6-months. *HRQoL* Health-related Quality of Life, *I*^*2*^ Higgin’s I^2^ statistic, *CI* confidence interval

### Participants

In total, 2064 participants were included in this review, with study sample sizes ranging from 40 [91] to 500 [94]. Most studies (*n* = 12) included more women than men and two studies included only women [90, 96]. Participants’ primary diagnoses were cancer (*n* = 7) [80, 89, 93–97], diabetes type II (*n* = 4) [84, 88, 90, 91], chronic heart disease (*n* = 1) [78], chronic obstructive pulmonary disorder (*n* = 1) [84], multiple sclerosis (MS) (*n* = 1) [83], epilepsy (*n* = 1) [92], and chronic kidney disease (*n* = 1) [82] (non-exclusive categories as some studies focused on two disease groups). Mean reported age in the sample ranged from 35.0 to 70.8 years. Depressive symptomatology across study groups ranged from mild to severe, with the mean reported symptoms most often in the moderate range [41–44, 47–49, 98, 99].

### Interventions

Included interventions are described in Table 2. Six of the 15 studies evaluated the same or similar interventions in different populations (4 one type of intervention and 2 another) [83, 92–95, 97]. Time spent participating in interventions (duration) ranged from 20 [88] to 2340 min [96] (*n* = 14, mean = 552.9, SD = 662.9). The length of time over which interventions were delivered ranged from one session [88] to two 12 month programs [89, 96].

The primary format of the interventions was individual (*n* = 11) [78, 80, 83, 84, 88, 91–95, 97]; however, three studies used a group format [82, 90, 96], and one included both group and individual sessions [89]. In terms of mode of delivery, seven studies used a combination of face-to-face and telephone contact (most favoured face-to-face contact with telephone follow-up only if needed) [78, 91, 93–97], four were delivered entirely face-to-face [82, 84, 89, 90], three were online [80, 83, 92], and one was a video on a computer tablet [88].

In terms of level of guidance, most were led by an interventionist (*n* = 11) [78, 82, 84, 89–91, 93–97], three were self-directed (two online programs and one video) [83, 88, 92], and one intervention was guided self-directed (participants independently worked through the intervention with feedback on exercises) [80]. The interventionists were all HCPs, other than one provided by a trained research assistant supervised by a psychologist [91]. Four of the interventions were delivered by nurses supervised or supported by psychiatrists [93–95, 97], three were delivered solely by nurses [78, 84, 90], one by psychologists [96], and the remaining two interventions were delivered by interdisciplinary HCPs [80, 82].

The content of the interventions focused on a combination of structured problem-solving (*n* = 12) [78, 80, 84, 89, 91–97], providing disease specific health information (*n* = 13; it was an optional component in two of the 13 interventions) [78, 82, 88–97], relaxation and stress management (*n* = 7) [82, 83, 88–90, 92, 96], using CBT principles (e.g., challenging negative self-talk) (*n* = 5) [83, 84, 89, 90, 92], care coordination (e.g., communicating the depressive symptoms to HCP team members) (*n* = 4) [93–95, 97], and finding health services (*n* = 2) [78, 88].

Eleven interventions were coded for a possible 13 depression self-management skills (interventions that contained the same content were combined). The skills identified for each intervention are summarized in Appendix A. Across the sample, the mean number of skills was 6.2 (SD = 2.4, range 2–11). The most frequently included skill was problem-solving (*n* = 9), followed by decision-making (*n* = 8), taking action (*n* = 7), social support (*n* = 7), and self-tailoring (*n* = 7). Less frequently addressed skills were social support (*n* = 1), resource utilization (*n* = 2), and forming partnerships with HCPs (*n* = 2).

### Methodological quality

Quality assessment scores are included in Table 2 and detailed scoring is available in Appendix D. The mean quality assessment score across the sample was 11.2 (SD 2.14) out of a possible 17 indicating that on average the studies were of moderate methodological quality. Scores ranged from 6 to 14 with four studies assessed as being of high methodological quality [91, 94, 95, 97]. The least met criteria related to blinding.

### Outcomes: Descriptive and meta-analysis

#### Primary outcome: Depression

**Fig. 2:**
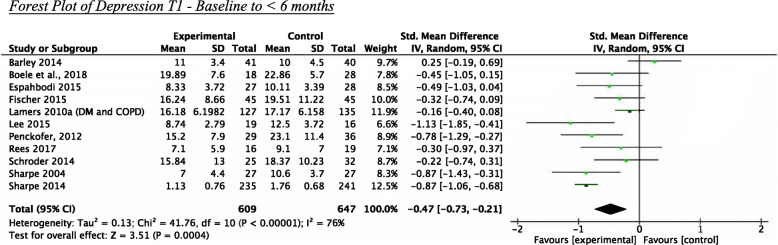
Forest Plot of Depression T1 - Baseline to < 6 months

Eleven studies were included in the meta-analysis for the T1 period (see Fig. 2). The pooled effect size of − 0.47 [95% CI -0.73, − 0.21] was significant with high heterogeneity I^2^ = 76% and favoured the interventions over the control conditions. The results remained significant after the Knapp-Hartung (KH) conversion [95% CI -0.74, − 0.02]. Statistically significant effect sizes ranged from − 0.78 [90] to − 1.13 [88].

When examining potential sources of heterogeneity, three studies reported pharmacological co-intervention that was significantly imbalanced between the intervention and control groups (participants in the intervention group were more likely to begin pharmacotherapy than those in the control group) [78, 93, 94]. When these studies were removed from the meta-analysis, the pooled effect size with 8 studies was of − 0.41 [95% CI − 0.61, − 0.20] with I^2^ = 32%. Using both the DerSimonian–Laird and the more conservative KH approach and examining potential sources of heterogeneity, the findings of all analyses indicated a statistically significant moderate effect of interventions as compared to control conditions. The two studies not included in the meta-analysis measuring depression outcomes reporting findings in line with those of the meta-analysis [95, 96].

**Fig. 3:**
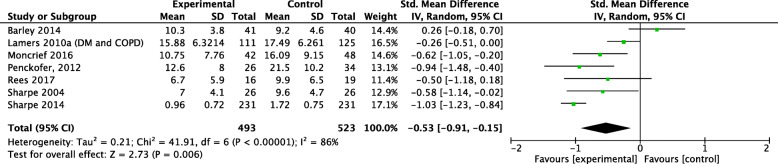
Forest Plot Depression T2 ≥ 6 months post-baseline

In the T2 time period, 7 studies were included in the meta-analysis (see Fig. 3). The pooled effect size of − 0.53 [95% CI -0.91, − 0.15] was statistically significant with high heterogeneity, I^2^ = 86% favouring the interventions. The results remained significant after KH conversion [95% CI -95, − 0.13]. Excluding the same three studies as in T1 [78, 93, 94] from the meta-analysis resulted in a pooled effect size, with 4 studies, of − 0.53 [95% CI -0.84, − 0.21] with moderate heterogeneity, I^2^ = 50%. The one study [97] not entered into the meta-analysis at T2 also favored the intervention over control group.

#### Secondary outcomes

A summary of results for secondary outcomes is presented in Table 3. Forest plots of meta-analysis results at the T1 and T2 periods for secondary outcomes are in Appendix E.

**Table 3:** Moderator analyses outcomes T1

Variables	# of studies	Pooled ES	L95	U95	***P***-value	I^**2**^	Meta-regression ***p***-value
**Overall**	8	− 0.41	− 0.61	− 0.20	< 0.001	32%	
**Disease**							
Cancer	1	−0.45	− 1.05	0.15			
Other	7	−0.41	− 0.65	− 0.18	0.001	41%	
**Baseline depression level**							0.926
Mild to moderate	5	−0.42	− 0.72	− 0.11	0.007	44%	
Moderately severe to severe	3	−0.43	−0.75	− 0.11	0.009	26%	
**Level of guidance**							0.840
Guided	5	−0.37	−0.62	− 0.13	0.002	26%	
Self-directed	3	−0.49	− 0.96	− 0.02	0.042	56%	
**Mode of delivery**							0.840
Face to face	5	−0.37	−0.62	− 0.13	0.002	26%	
Not face to face	3	−0.49	− 0.96	− 0.02	0.042	56%	
**Provider**							0.840
Professional	5	−0.38	−0.63	− 0.14	0.002	28%	
Self-directed	3	−0.5	− 0.98	− 0.02	0.042	58%	
**Format**							
Individual	6	−0.33	− 0.55	− 0.11	0.004	25%	
Group	2	−0.65	−1.01	− 0.28	0.001	0%	
**Duration of Intervention**							1.000
< 300 min	4	−0.47	− 0.85	− 0.09	0.016	58%	
≥ 300	4	−0.41	− 0.66	− 0.16	0.002	0%	
**Control Group**							
Active	2	− 0.70	−1.52	0.11	0.09	64%	
Not active	6	−0.33	− 0.51	− 0.15	< 0.001	10%	
**Methodological Quality**							
Low	1	−0.49	−1.03	0.04	0.072		
Moderate	7	−0.41	− 0.64	− 0.17	0.001	40%	
**Length of Intervention**							0.858
< 3 months	5	− 0.43	− 0.70	− 0.17	0.001	15%	
≥ 3 months	3	−0.41	−0.81	− 0.02	0.042	60%	
**Depression Self-Management Skills**							
**Decision-making**							0.016*
No	3	−0.75	−1.08	− 0.42	< 0.001	0%	
Yes	5	−0.23	−0.41	− 0.05	0.011	0%	
**Problem-solving**							
No	2	−0.90	−1.31	− 0.48	< 0.001	0%	
Yes	6	−0.26	−0.43	− 0.09	0.003	0%	
**Resource Utilization**							
No	7	−0.31	− 0.47	− 0.15	< 0.001	0%	
Yes	1	−1.13	−1.85	−0.41	0.002		
**Partnerships with HCPs**							
No	7	−0.49	−0.70	− 0.28	< 0.001	4%	
Yes	1	−0.16	− 0.4	0.08	0.197		
**Taking Action**							0.020*
No	3	−0.75	−1.08	− 0.42	< 0.001	0%	
Yes	5	−0.23	−0.41	− 0.05	0.011	0%	
**Behavioural Activation**							1.000
No	4	−0.47	−0.85	− 0.09	0.016	58%	
Yes	4	−0.41	−0.66	− 0.16	0.002	0%	
**Cognitive Restructuring**							0.297
No	3	−0.61	−1.06	− 0.16	0.008	34%	
Yes	5	−0.32	−0.53	− 0.11	0.003	21%	
**Self-monitoring**							0.694
No	5	−0.45	−0.71	− 0.19	0.001	12%	
Yes	3	−0.38	−0.78	0.02	0.065	57%	
**Health Habits**							0.283
No	4	−0.25	−0.45	−0.05	0.014	0%	
Yes	4	−0.56	−0.93	− 0.19	0.003	49%	
**Communicating about Depression**							
No	7	−0.34	− 0.53	−0.14	0.001	17%	
Yes	1	−0.78	−1.29	−0.27	0.003		
**Social Support**							0.279
No	3	−0.23	−0.43	−0.02	0.036	0%	
Yes	5	−0.53	−0.82	− 0.23	< 0.001	33%	
**Relaxation**							0.212
No	3	−0.21	− 0.42	0.00	0.053	0%	
Yes	5	−0.53	− 0.82	− 0.25	< 0.001	32%	
**Self-tailoring**							0.747
No	4	−0.47	− 0.80	− 0.14	0.005	34%	
Yes	4	−0.37	−0.67	− 0.07	0.015	40%	
**Number of Skills**							0.311
1–6	4	−0.56	−0.88	− 0.24	0.001	6%	
7–13	4	−0.32	−0.57	− 0.07	0.013	37%	

Random effect model was used to compute the pooled effect size.

*p < 0.05 indicating statistical significance

##### Anxiety.

In the T1 period, 7 studies were entered in the meta-analysis [78, 82, 86, 88, 90, 93, 94]. The pooled effect size was − 0.42 [95% CI -0.73, − 0.12] with heterogeneity of I^2^ = 73% in favour of the interventions. This finding remained significant after HK conversion [95% CI -0.82, − 0.02]. Removing the same three studies as identified for depression due to high pharmacological co-intervention [78, 93, 94], the pooled effect size was significant, − 0.29 [95% CI -0.53, − 0.06], with no heterogeneity, I^2^ = 0%. One study [95] was not entered into the meta-analysis and favoured the intervention.

For the T2 time period, 4 studies were included in the meta-analysis [78, 90, 93, 94]. The pooled effect size was − 0.52 [95% CI -0.94, − 0.10] with high heterogeneity, I^2^ = 77%. This was not significant after HK conversion. The one study [97] not entered into the meta-analysis reported a significant improvement in anxiety in the intervention group as compared to the control.

##### Health-related quality of life.

*Mental component score (MCS) of health-related quality of life*. Five studies were included in the meta-analysis in the T1 period [78, 80, 86, 88, 90]. The pooled effect size was statistically significant with moderate heterogeneity, 0.43 [0.09, 0.76] with I^2^ = 60%. However, it was not significant after HK conversion. Removing the results reported by Barley et al., 2014 [78] did not improve heterogeneity. For T2, the 3 studies were entered in the meta-analysis and did not result in significant pooled effect sizes [84, 90, 91].

*Physical component score. (PCS) of health-related quality of life* Five studies were entered into the meta-analysis for the T1 period [78, 80, 84, 88, 90] and 3 studies for the T2 time period [78, 84, 90]. The pooled effect sizes were not significant at either time point.

##### Fatigue.

For the T1 period, three studies were included in the meta-analysis [80, 83, 94] and resulted in a significant pooled effect size of − 0.36 [− 0.67, − 0.06] with moderate heterogeneity, I^2^ = 50%. The results were not significant if Sharpe, Walker [94] was removed from the analysis. For T2, only one study reported the needed data for meta-analysis [94] and the results were statistically significant with an effect size of − 0.60 [− 0.78, − 0.41] in favour of the intervention. Of the three studies not included in the meta-analysis, two reported in favour of the intervention at the T1 and T2 time periods [95, 96], and the remaining study reported no significant effect on this outcome [97].

##### Glycemic control (HbA1c).

Three studies were entered into the meta-analysis in the T1 period [87, 90, 91] and the results of the pooled effect size were not significant. At T2, 4 studies were included in the meta-analysis [87, 89–91] and the results were significant with an effect size of − 0.35 [CI 95% -0.62, − 0.07] and no heterogeneity, I^2^ = 0%.

#### Moderator analyses

The results of the moderator analyses for the T1 time period are presented in Table 3. The three studies found to be outliers [78, 93, 94] due to imbalanced pharmacological co-intervention were not included in these analyses. There was not enough data to perform meta-regression for the T2 period (4 studies total); however, 8 studies were included at T1. Meta-regressions were performed for the following 5 moderators, as there were 4 studies in each sub-group: duration of the intervention (< 300 min or ≥ 300 min), behavioural activation (yes/no), health habits (yes/no), self-tailoring (yes/no), and number of self-management skills included in the intervention [1–6 or 7–13]. None were found to be significant. Additional meta-regressions were run for the following 9 moderators including a minimum of 3 studies per sub-group: baseline depression level of the study sample (mild to moderate/moderately severe to severe), level of guidance (guided/self-directed), intervention provider (professional/self-directed), length of the intervention (< 3 months/ ≥ 3 months), decision-making (yes/no), taking action (yes/no), cognitive restructuring (yes/no), self-monitoring (yes/no), and relaxation (yes/no). Of these, the results were significant only for the two self-management skills of decision-making (*p* = 0.020) and taking action (*p* = 0.017).

## Discussion

To our knowledge, this is the first systematic review to examine the effect of self-management interventions on reducing depressive symptoms among adults with chronic physical disease(s) and co-occurring depression. The results were drawn from the findings of 15 studies. Meta-analysis was conducted for two time periods for the primary outcome of depression as well as the secondary outcomes of anxiety, health-related quality of life (mental component and physical component), fatigue, and glycemic control. Analyses of potential moderators of intervention effect on the primary outcome were performed to identify active elements. Overall, the findings support: a) an effect of interventions on improving depression and anxiety as well as glycemic control at ≥ 6-months post-baseline; b) intervention duration and intervention length were not found to impact effect; and c) some moderators merit further attention in future studies, including level of guidance and type of self-management skills included.

### Effect on participant outcomes

The results of the review indicate a moderate effect of self-management interventions on depression and a small effect on anxiety. These results are consistent with the broader literature on self-management for individuals with depression or chronic physical diseases. A systematic review by Houle et al. (2013) found that self-management interventions reduced depressive symptomatology in the general adult population and improved functioning, self-efficacy, and self-management behaviours [65]. Findings related to relapse of depression were mixed. The results of the present review could not shed further light on this issue, as only three studies reported intervention and control group data at 12 or more months [78, 89, 93]. Another recent systematic review assessing the effect of face-to-face self-management interventions for adults with a chronic diseases (not necessarily with co-morbid depression) found that efficacious interventions were more likely to include psychological coping or stress management strategies [27].

Results of the present review can also be compared to those of a review of psychotherapy for adults with depression (with or without chronic co-morbid chronic conditions) that found the interventions to have a larger but still moderate effect size (*d* = 0.68) in improving depressive symptoms [100]. Reviews of the effect of psychotherapy on co-morbid depression in adults with chronic physical diseases report between small and large effect sizes [101]. Due to the relatively small number of studies, strong comparisons or conclusions cannot be drawn; however, results suggest that depression self-management interventions for this population may have a similarly beneficial effect while generally being more cost-effective.

### Moderator analyses

The analyses of moderators found no significant difference in intervention effect on depression based on intervention length or duration. These findings have important implications for the integration of such interventions into resource constrained clinical environments. Seeking evidence-based cost- and time-effective interventions is imperative for the sustainability of providing these in clinical practice [27]. As feasibility is a priority, further investigation of these intervention characteristics is warranted.

Self-directed interventions (no contact with an HCP or coach) did not have significantly lower effect sizes than guided ones. However, this must be interpreted with much caution as the sample size was very small. A number of reviews support the efficacy of minimally guided and self-directed interventions [102–105, 120]. A review of self-directed psychological interventions found a small significant effect of interventions (*d* = 0.23 at post-test, *d* = 0.28 from 4- to 12-months) on depression [121]. Only one trial to our knowledge has directly compared the effects of a guided (coached) and self-directed depression intervention for adults with chronic conditions [106]. This trial was excluded from our meta-analysis because of the active control group. The results indicated an overall significant benefit of coaching on depressive symptoms at 3 months; among those who were not receiving psychological treatment at study entry, the benefit was extended to 6 months. The increased effect is likely explained by the greater adherence to the self-care tools in the coached (guided) group [22, 103].

The results indicated that not all self-management skills may be equally beneficial in improving depressive symptoms. Of the 13 self-management skills examined, two of them, decision-making and taking action, were potentially significant moderators of the primary outcome of depression. Of the skills examined, six, drawn from the work of Lorig & Holman (2003), are considered “core” self-management skills that are applicable across chronic diseases. The remaining seven skills were drawn from the literature specifically on the self-management of depression. The findings of this study parallel those of a previous review of non-pharmacological depression interventions for caregivers, which found that problem-solving, decision-making, and taking action were significant moderators of depression [70]. It is notable that neither review identified depression specific skills as significant moderators. Interestingly, a review of self-management interventions in a different population, adults with low income or low health literacy, also found that problem-solving and taking action were more often included in efficacious interventions [69]. Due to limited data, it was not possible to examine problem-solving in this review. Together, the findings of these reviews suggest that developing core self-management skills to foster behaviour change might be more important than disease-specific self-management skills.

### Methodological quality

The majority of studies were of moderate methodological quality, and none met all of the criteria. Due to insufficient sample size, it was not possible to examine the heterogeneity among studies of different methodological quality. Adherence rates were reported in nine of 15 studies, similar to a previous review of self-care interventions for anxiety or depression that found 55% of studies reported adherence measures [107]. A number of studies described the amount of time participants engaged in the intervention; however, no study indicated a minimal therapeutic dose or exposure to the intervention. More detailed standardized measures of adherence including activity or module completion, time spent, and active engagement have been proposed to address this issue [108]. The pre-established criteria, based on Cochrane Risk of Bias tool, were difficult to apply to self-directed interventions. For example, intervention fidelity becomes essentially synonymous with adherence in the case of self-directed interventions. In this case, blinding of participants to group allocation may be of greater importance as they are in essence the interventionists and frequently self-report their own outcomes.

If attrition was 20% or less across the sample, the criterion was assessed as being met [71, 109]. However, in six of the 15 studies, attrition rates were notably higher in the intervention group as compared to the control [78, 84, 89–92] and no studies reported greater attrition in the control group. This raises concern regarding the potential impact of attritional bias on study outcomes [110]. Though four of these studies used intention-to-treat analyses, this may not entirely mitigate the impacts of this missing data [111]. Reporting the baseline characteristics of participants who were and were not included in analyses is recommended to help address this [111]. Further, previous work has addressed predictors of attrition of participants with depression from pharmacological trials, but this has not been thoroughly addressed in psychosocial trials [112, 113].

### Reported outcomes

There was substantial variety in the outcomes reported across studies and measurement instruments used. Of the 38 outcomes measured across studies, only five were reported at least three times at one time point. Further, six instruments were used to measure the primary outcome of depression. These instruments were also used to establish the presence of at least mild symptoms of depression across the sample, a criterion for inclusion in this review. This is notable as a recent co-calibration study examining the variations in five commonly used depression self-report instruments found that the cut-off scores across scales were not equivalent [114]. The impact of this on the primary outcome could have resulted in an under- or over-estimation of intervention effect.

### Strengths and limitations

The study methods were guided by the Cochrane Handbook and the PRISMA statement, and the protocol was registered with PROSPERO [38, 39]. The methods were outlined in detail and are reproducible. Given some terminological ambiguity in the literature regarding what constitutes a self-management intervention, another strength of this review was the search terms applied were very inclusive. Interventions that were not self-described as self-management were included in the review based on the meeting the predetermined definition based on current self-management literature. This is in line with recommendations by Lorig and Holman (2003). However, due to the variations in definitions of self-management, it is possible that other teams would have identified different interventions for inclusion. Further, self-management interventions are recommended as part of a stepped care approach to depression management in which a sequence of treatments are provided based on an individual’s response [115]. Lower intensity interventions, such as self-management, are initially delivered and treatment is ‘stepped up’ to higher intensity psychological interventions (e.g., pharmacotherapy, psychotherapy) for those who do not benefit from the initial treatment [22, 105, 115, 116]. Though four such interventions were screened at the full text stage, none were included as they did not meet the a priori inclusion criterion of providing non-therapeutically active control group data. Future research could evaluate the role of self-management within the structure of stepped care approach to intervention delivery.

The results should be interpreted with caution as the sample size was small with substantial heterogeneity, though this was found to be largely attributable to the confounding impact of pharmacotherapy. The limited number of studies prevented further examination of sources of heterogeneity and analyses of moderators was also conducted with a very small sample. There was only sufficient data to examine outcomes up to 6 months; longer-term outcome data is needed. Further, most of the included studies were focused on those with cancer or diabetes. Though the findings offer potential future avenues for exploration, further evidence is required to investigate longer-term outcomes, sources of heterogeneity, and possible differences in chronic physical disease populations.

## Conclusion

This is the first systematic review to examine the effect of self-management interventions on depression in adults with co-occurring chronic physical diseases. The findings indicate the interventions reduced depression with a moderate effect size and anxiety with a small effect size. Impact of the interventions on other psychosocial and physical health outcomes was mixed. Recommendations include further evaluation of the impact on the amount of guidance, length, and duration of interventions, as self-directed, shorter and thereby less resource intensive interventions may be effective. Including self-management skills of decision-making and taking action in future interventions is also recommended.

## Figures and Tables

**Fig. 4:**
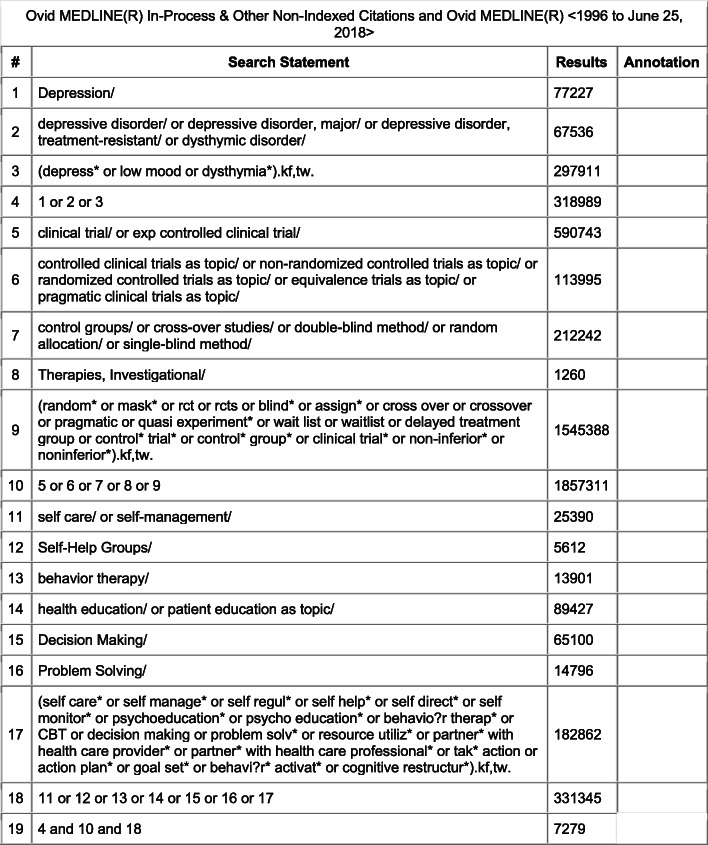
Sample Search Strategy from Ovid Medline conducted June 25th, 2018

**Fig. 5:**
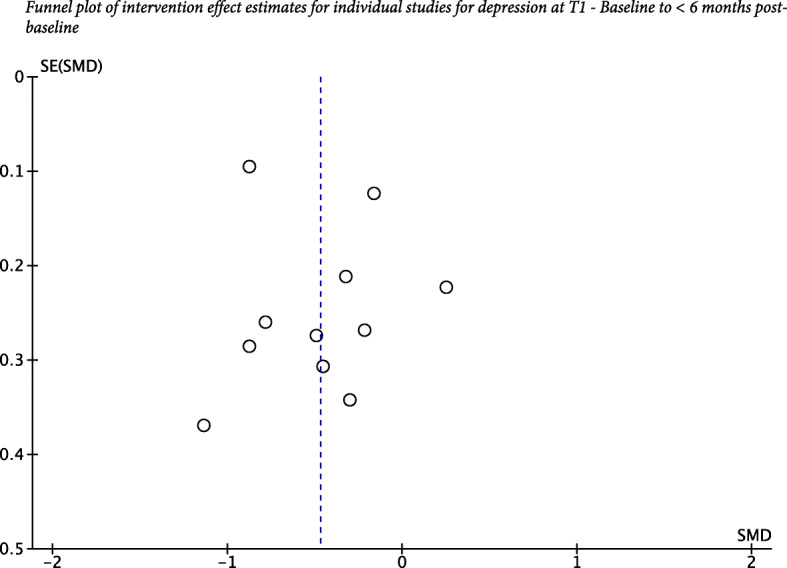
Funnel plot of intervention effect estimates for individual studies for depression at T1 - Baseline to < 6 months

**Fig. 6:**
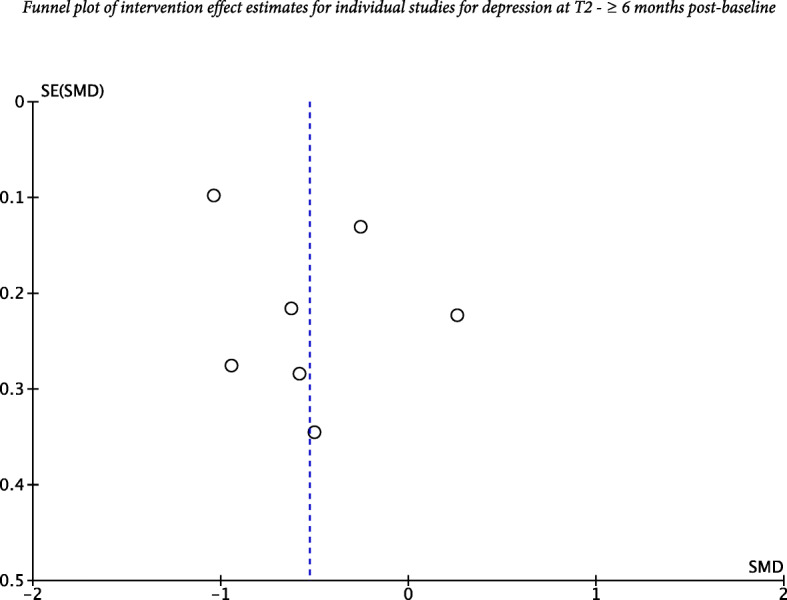
Funnel plot of intervention effect estimates for individual studies for depression at T2 - ≥ 6 months post-baseline

**Fig. 7:**
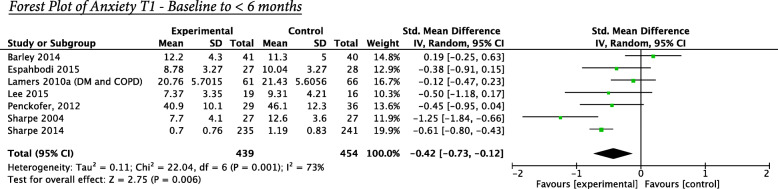
Forest Plot of Anxiety T1 - Baseline to < 6 months

**Fig. 8:**
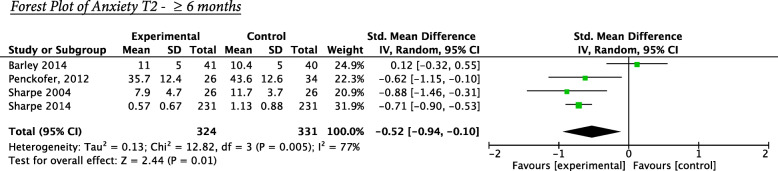
Forest Plot of Anxiety T2 ≥ 6 months post-baseline

**Fig. 9:**
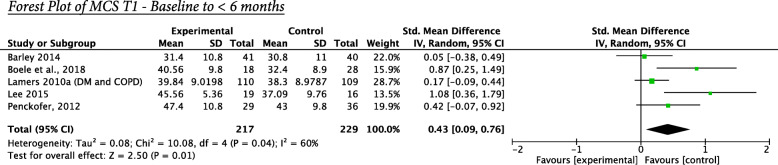
Forest Plot of MCS T1 - Baseline to < 6 months

**Fig. 10:**
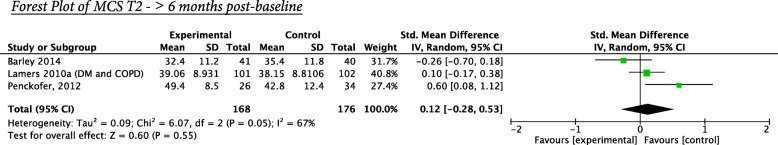
Forest Plot of MCS T2 ≥ 6 months post-baseline

**Fig. 11:**
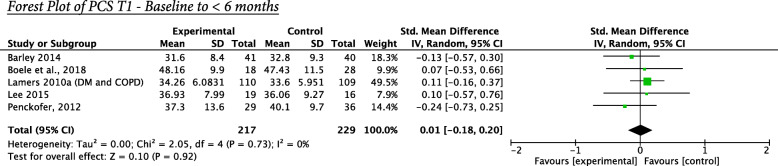
Forest Plot of PCS T1 - Baseline to < 6 months

**Fig. 12:**
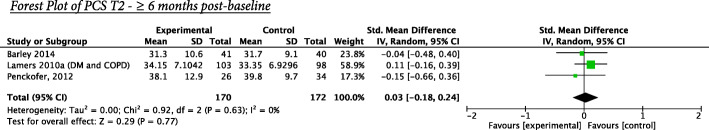
Forest Plot of PCS T2 ≥ 6 months post-baseline

**Fig. 13:**
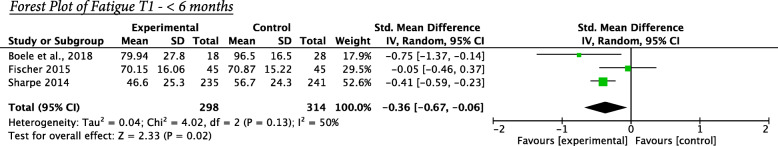
Forest Plot of Fatigue T1 - Baseline to < 6 months

**Fig. 14:**
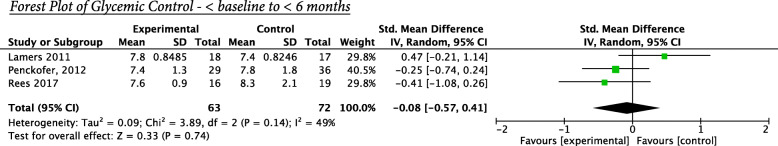
Forest Plot of Glycemic Control T1 - Baseline to < 6 months

**Fig. 15:**
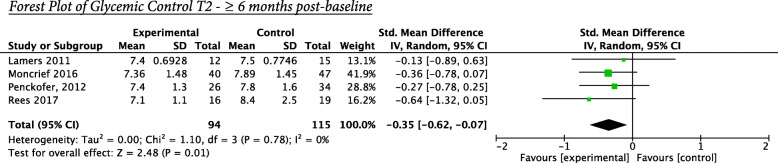
Forest Plot of Glycemic Control T2 ≥ 6 months post-baseline

## Data Availability

The datasets used and/or analysed during the current study are available from the corresponding author on reasonable request.

## Appendix 1

**Table 4:** Depression self-management skills included in the interventions

	Barley et al. (2014) [78]	Boele et al. (2018) [80]	Espahbodi et al. (2015) [82]	Fischer et al. (2015) [83] & Schroder et al. (2014)* [92]	Lamers et al. (2010) [86]	Lee et al. (2014) [88]	Moncrief et al. (2016) [89]	Penckofer et al. (2012) [90]	Rees et al. (2016)	Sharpe et al. (2004) [93], [93] Sharpe et al. (2014) [94], Strong et al. (2008) [95], & Walker et al. (2014)† [97]	Thorton et al. (2009) [96]	Total
**Decision-Making** Often occurs in the context of problem-solving and is based on having enough and appropriate information to meet common changes associated with chronic illness [25].	1	1	0	1	1	0	1	0	1	1	1	8
**Problem-solving** Using a structured approach and learning skills such as problem definition, generating solutions, implementation, and evaluation of results to move towards a solution [25, 29].	1	1	1	1	1	0	1	0	1	1	1	9
**Resource Utilization** Learning how to seek out many resources (using different sources) [25].	1	0	0	0	0	1	0	0	0	0	0	2
**Forming Partnerships with HCPs** Learning how to provide disease-related feedback to HCPs and make informed treatment decisions and discuss with HCPs [25].	0	0	0	0	1	0	1	0	0	0	0	2
**Taking Action** Making a plan and carrying it out, learning skills involved in behaviour change [25].	1	1	0	1	1	0	1	0	1	1	0	7
**Behavioural Activation** Learning to gradually increase positive activities through effective goal setting [29]	0	0	0	1	0	0	1	1	1	0	0	4
**Cognitive Restructuring** Learning to identify depressive self-talk, challenge it, and come up with fair-realistic ways of evaluating situations [29].	0	1	0	1	1	0	1	1	0	0	0	5
**Self-Monitoring** Monitoring depression symptoms and evaluating whether current strategies are working effectively and, when necessary, reassessing treatment plans [63].	1	0	0	0	1	0	1	1	1	0	0	5
**Health Habits** Learning about the links between health habits (e.g., sleep, diet) and mental health. Learning how to enact helpful health related habits [64, 65].	0	0	0	1	0	1	1	1	0	0	1	5
**Communicating about Depression** Learning to explain what it means to experience depression to family members, friends, and colleagues [117].	0	0	0	0	0	0	0	1	0	0	0	1
**Social Support** Arranging instrumental and emotional support [117–119]. Involving close friends/family in treatment and support [117, 118].	1	1	0	1	0	1	1	1	0	0	1	7
**Relaxation** Maintaining or developing activities related to relaxation (e.g., meditation, breathing exercises).	0	0	1	1	0	1	1	1	0	0	1	6
**Self-tailoring** Learning to use other self-management skills based on a personal evaluation of your own needs.	1	1	0	0	1	0	1	1	1	1	0	7
**Total**	7	6	2	8	7	4	11	8	6	4	5	

***Fischer et al. (2015) and Schroder et al. (2014) delivered the same intervention to different disease populations. **†** Intervention delivered by Sharpe et al. (2004), Sharpe et al. (2014), Strong et al. (2008), & Walker et al. (2014) identically reported in terms of content related to self-management skills. Delivered to different oncology samples and change reported in the interventionists

## Appendix 4

**Table 5:** Quality Assessment of Included Studies

	Barley et al., 2014 [78]	Boele et al., 2018 [80]	Espahbodi et al., 2015 [82]	Fischer et al., 2015 [83]	Lamers et al., 2010a b [88. 100]; 2011 [87]	Lee et al., 2014 [88]	Moncrief et al., 2016 [89]	Penckofer et al., 2012 [90]	Rees et al., 2017 [91]	Schröder et al., 2014 [92]	Sharpe et al., 2004 [93]	Sharpe et al., 2014 [94]	Strong et al., 2008 [95]	Thornton et al., 2009 [96]	Walker et al., 2014 [97]	Total
**Inclusion criteria specified**	1	1	1	1	1	1	1	1	1	1	1	1	1	1	1	15
**Pre-specified primary and secondary outcomes**	1	1	1	1	1	1	1	1	1	1	1	1	1	1	1	15
**Psychometric properties provided**	0	1	1	0	0	0	1	1	1	1	1	1	1	1	1	11
**Explicit power calculation**	0	1	0	1	1	1	1	1	0	1	1	1	1	0	1	11
**Target sample size reached**	0	0	0	0	1	1	0	0	1	0	1	1	1	0	0	6
**Randomization method specified and truly random**	1	1	0	1	1	0	0	1	1	1	0	1	1	1	1	11
**Randomization - Allocation concealed**	1	1	0	1	1	0	1	0	1	0	0	1	0	0	1	8
**Potential co-interventions discussed**	1	0	0	0	1	1	0	1	1	1	1	1	1	0	1	10
**Outcome assessors blind**	1	0	0	0	0	1	1	0	1	0	1	1	1	1	1	9
**Participants blind to treatment allocation**	0	0	0	0	0	1	0	0	0	0	0	0	0	0	0	1
**Interventionists blind**	1	0	0	0	0	1	0	0	0	0	0	0	0	0	0	2
**Adherence > 75%**	0	0	0	1	1	1	0	1	1	1	1	1	1	1	1	11
**Intention-to-treat data analysis**	0	1	0	1	1	0	1	1	0	1	0	1	1	1	1	10
**Overall > 80% of sample in primary data analysis**	1	0	1*	0	0	1	0	1	1	0	1	1	1	1	1	10
**Reason for attrition stated**	1	1	1*	0	1	1	1	1	1	0	1	0	1	1	0	11
**Baseline characteristics for both groups reported**	1	1	1	1	1	1	1	1	1	1	1	1	1	1	1	15
**Fidelity monitoring**	1	1	0	1	1	1	0	1	1	1	1	1	1	1	1	12
**Total (/17)**	**11**	**10**	**6**	**9**	**12**	**12**	**9**	**12**	**13**	**10**	**12**	**14**	**14**	**11**	**13**	

**Notes:** Score of 1 if criterion met; 0 if not met or information unavailable. Pilot studies given automatic 0 for explicit power calculation as aim of pilot studies is not establish efficacy or effectiveness. Pilot studies given 1 for target sample size reached if rationale was provided for sample size and this was met. Thornton et al. (2009) was a sub-group analysis of a larger trial. A post-hoc power calculation was not included; as such, this study was scored as 0 for both power calculation and target sample size reached. Randomization- allocation concealment criterion was met if the person conducting randomization was independent from the research team (e.g., data collection, analysis, development of project aims). For self-directed interventions, participants themselves were considered to the be outcome assessors and interventionists; as such, if participants in self-directed interventions were aware of their group allocation, outcome assessors and interventionist were deemed not to be blind and given a 0 for these criteria. Criterion of overall > 80% of sample in primary data analysis was based on the stated primary data collection time point. If no primary time point was specified, the first data collection point was used. Reasons for attrition stated was based on dropouts after randomization. For self-directed interventions, if adherence criterion was met, fidelity criterion was also considered to be met. Studies were considered to be of high methodological quality if 13–17 criteria were met, moderate quality if 8–12 were met, and low fewer than 8 were met.*Espahbodi et al. (2015): Those who did not complete all intervention sessions were excluded from the study

## Abbreviations

BDI-II: Beck depression inventory-II
BDI: Beck depression inventory
C: Control condition
CBT: Cognitive behavioural therapy
CES-D: Centre for epidemiological studies-depression
CHD: Coronary heart disease
CI: Confidence interval
CNS: Central nervous system
COPD: Chronic obstructive pulmonary disease
DSM: Diagnostic and statistical manual of mental disorders
ER: Emergency room
ES: Effect size (hedge’s g calculated at 95% confidence level)
HADS-D: Hospital anxiety and depression scale-depression
HbA1c: Hemoglobin A1c test for glycemic control
HCP: Healthcare professional
HRQoL: Health related quality of life and includes
PCS: Physical health composite scale
MCS: Mental health composite scale
ICD: International classification of diseases
IQR: Interquartile range
KH: Knapp-Hartung
MS: Multiple sclerosis
PHQ-9: Patient health questionnaire
PRESS: Peer Review for electronic search strategies
PRISMA: Preferred reporting items for systematic reviews and meta-analyses
PST: Problem-solving therapy
QoL: Quality of life
RCT: Randomized controlled trial
SD: Standard deviation
T < C: control superior to treatment
T = C: no significant differences between treatment and control conditions
T > C: treatment significantly superior to control
T: Treatment condition
WLC: Waitlist control group
